# Double “full moon” CTO plaque detected by computed tomography could predict high‐grade debulking techniques: A case‐report

**DOI:** 10.1002/ccr3.7325

**Published:** 2023-05-18

**Authors:** Giuseppe Panuccio, Carsten Skurk, Ulf Landmesser, Youssef S. Abdelwahed

**Affiliations:** ^1^ Department of Cardiology, Angiology and Intensive Care Medicine Deutsches Herzzentrum der Charité Berlin Berlin Germany; ^2^ Department of Medical and Surgical Sciences Magna Graecia University Catanzaro Italy; ^3^ DZHK (German Centre for Cardiovascular Research) Berlin Germany; ^4^ Berlin Institute of Health (BIH) Berlin Germany

**Keywords:** chronic total occlusions, complex PCI, computed tomography, coronary imaging, full‐moon calcifications, rotational atherectomy

## Abstract

Circular heavily calcified “Full Moon” plaques relevance for CTO‐PCI remains unclear. This case shows a patient with double “Full Moon” plaques‐CTO. Cardiac tomography identified these lesions and allowed to provide adequate debulking equipment. “Full Moon” plaques could predict CTO‐PCI complexity. CT can identify these lesions and help planning CTO‐PCI for increasing success rates.

## 1 INTRODUCTION

There is an increasing incidence of chronic total coronary occlusions (CTO). CTO are complex coronary lesions and known to represent a technical challenge for percutaneous recanalization procedures. However, the success rates of CTO‐percutaneous coronary interventions (CTO‐PCI) have increased tremendously with the continuous innovations in equipment and procedural techniques. Circular heavily calcified “Full Moon” like‐plaques have been observed in CTO patients; however, their frequency and relevance for CTO‐PCI outcomes remain unclear, despite they can be straightforward identified by coronary computed tomographic angiography (CCTA) during CT‐scans to select the CTO‐PCI recanalization strategy.

## 2 CASE HISTORY

**FIGURE 1:**
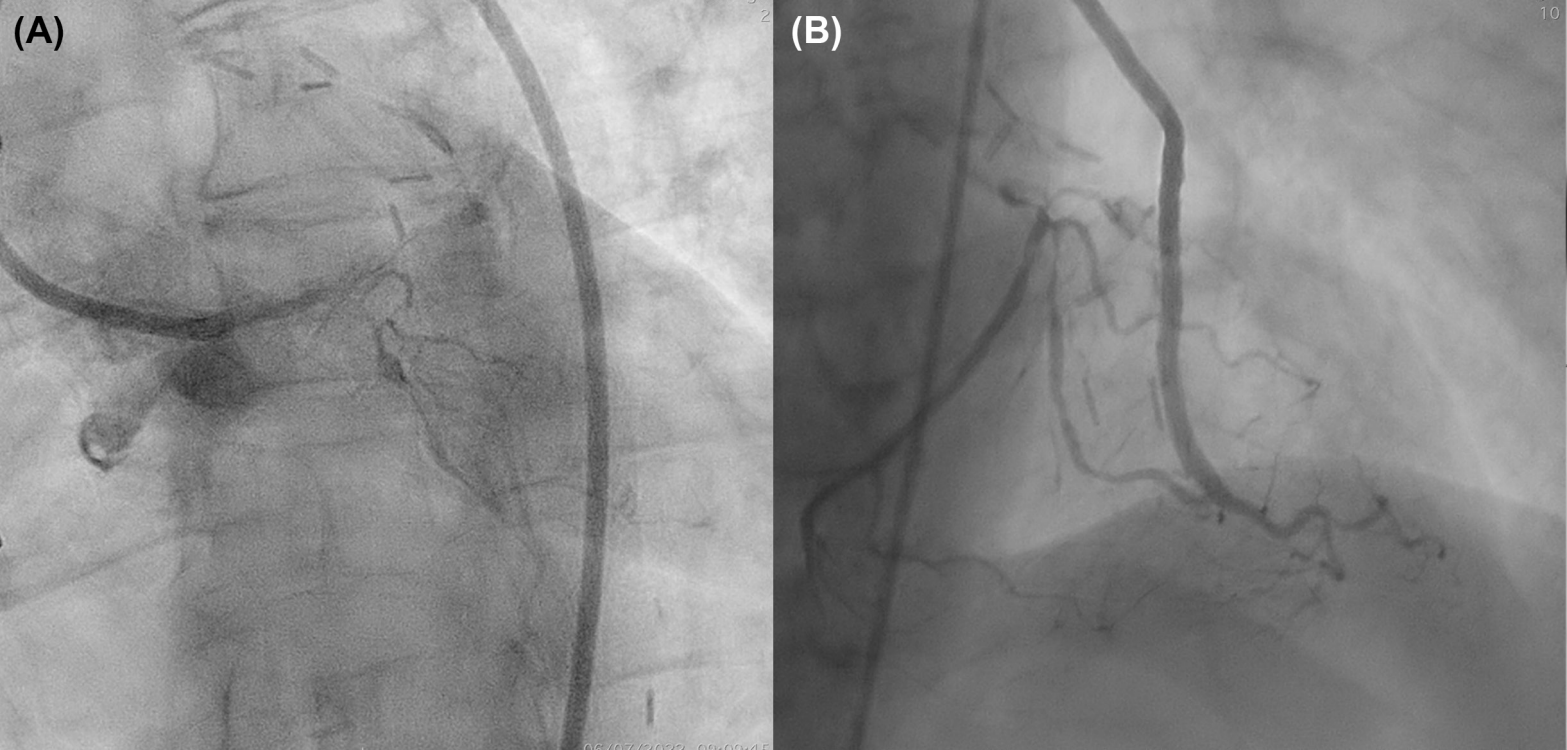
Baseline angiography (A, B).

We report a case of a 69‐year‐old female patient, admitted to our institution due to recurrent angina and positive stress test. The patient underwent coronary artery bypass grafting (CABG) 20 years before with a left internal mammary artery (LIMA) on left anterior descending (LAD) artery, a venous graft (VG) on the first diagonal branch (ID), and another VG on obtuse marginal branch (OM). Coronary angiography showed a patent VG‐OM but a significant stenosis in OM at the site of the bifurcation with the left circumflex artery (LCX, Figure 1A,B), considered as the cause of ischemia. Therefore, we decided to treat the LCX chronic total occlusion (CTO).

## 3 OUTCOME

**FIGURE 2:**
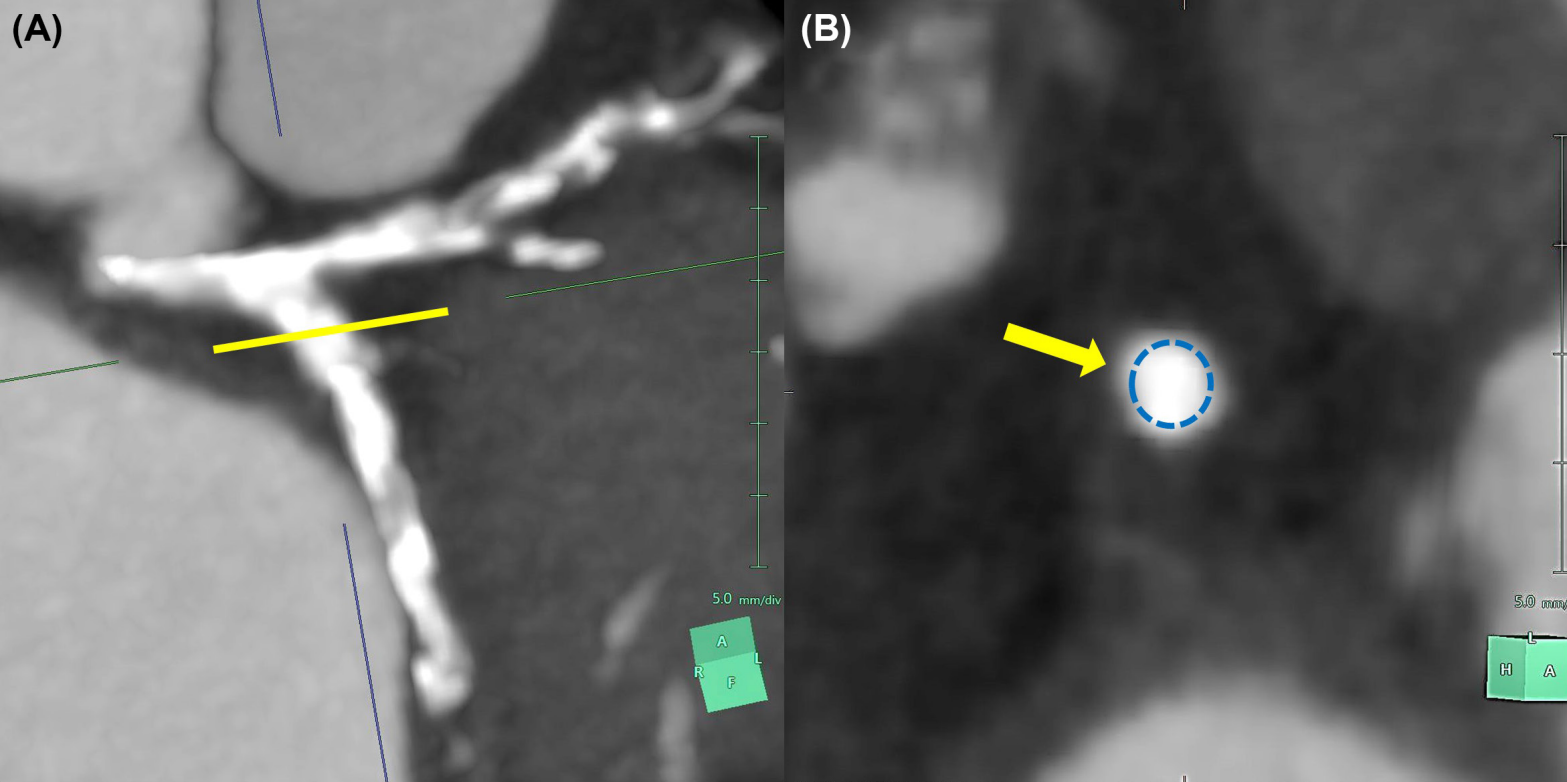
Yellow arrow pointing to the maximum point of the calcified plaque seen on MSCT using the slice view method (A), and its cross‐sectional image (B), revealing a fully obliterated lumen with “full moon”‐like appearance (blue dotted circle) in proximal LCX.

**FIGURE 3:**
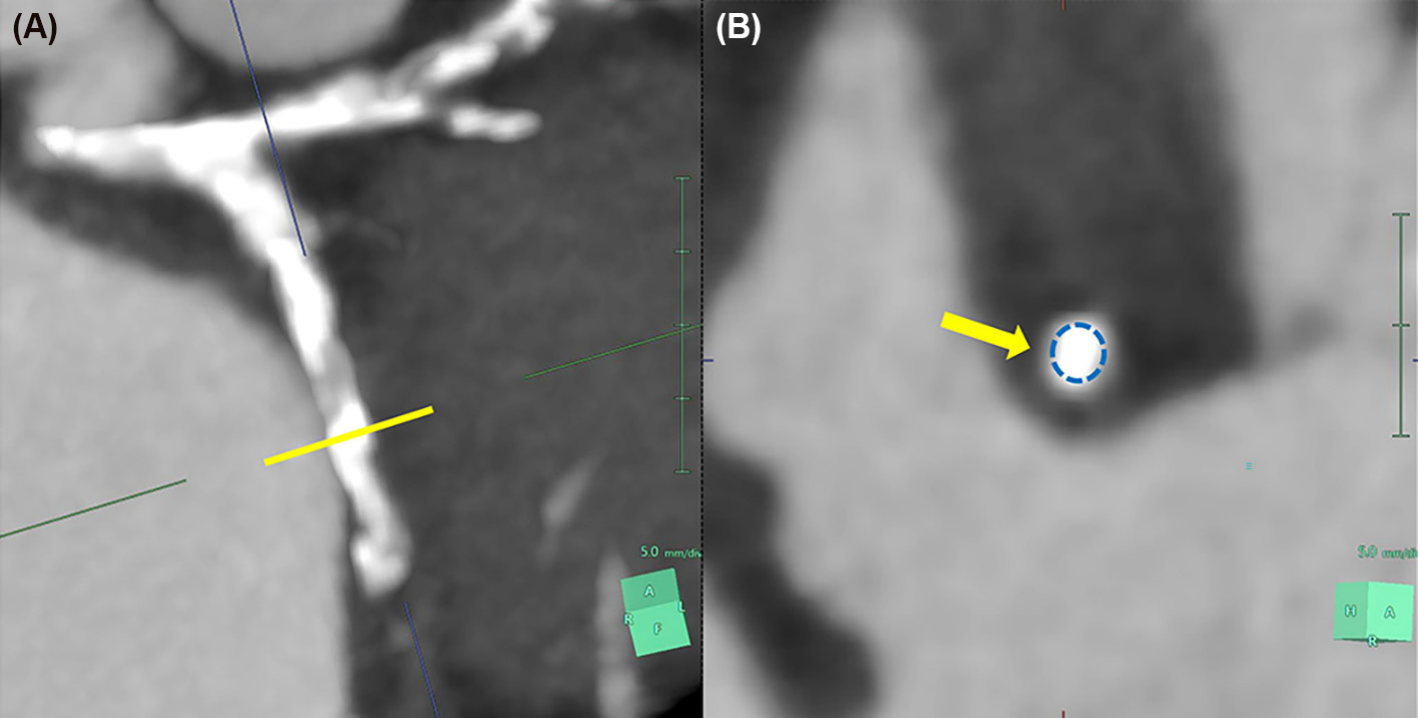
Second “full‐moon”‐like lesion in middle LCX (A‐B).

**FIGURE 4:**
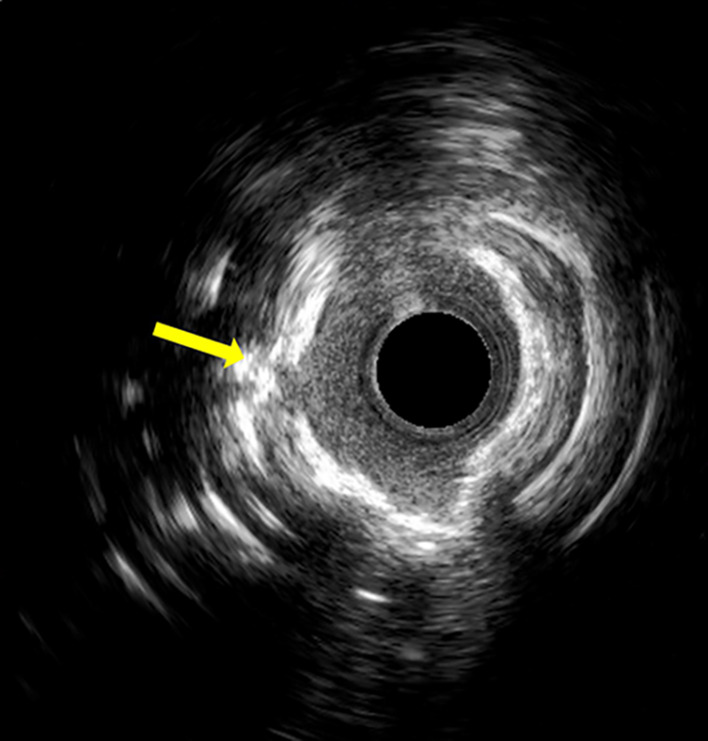
Yellow arrow pointing to crack within a heavily calcified plaque after rotablation.

**FIGURE 5:**
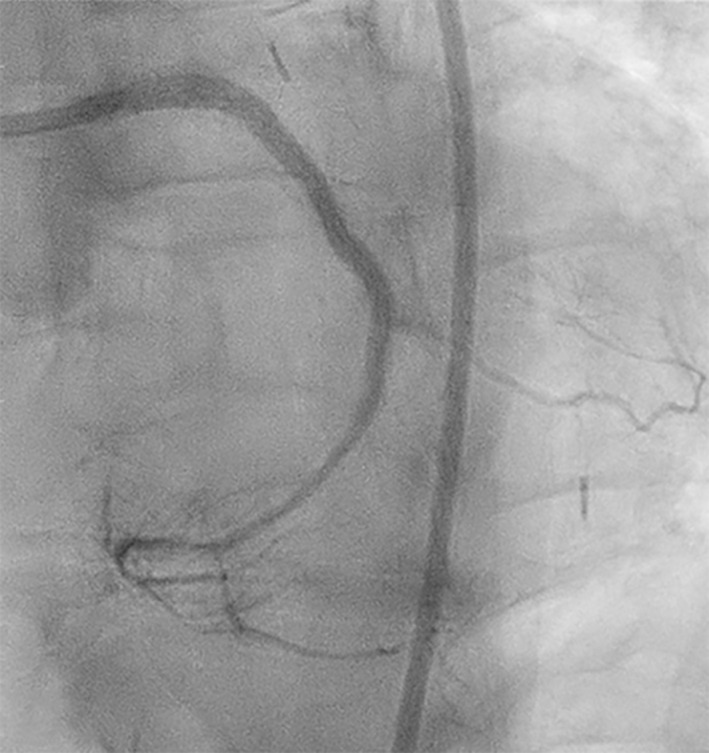
Final result.

Multislice computed tomography (MSCT) was performed before the LCX CTO‐PCI procedure, and it showed two complex plaques with a “full‐moon” morphology at the proximal and middle part of the vessel (Figures 2A,B and 3A,B). Therefore, a very complex procedure requiring intense debulking techniques was expected. An antegrade wire escalation approach (AWE) was decided to recanalize the vessel. With extreme difficulty, a stiff CTO guidewire (Gaia 3), was placed into the OM. However, despite several dilatations with small balloons (0.85 mm, 1.0 mm, and 2.0 mm) no significant antegrade flow was obtained, and it was not possible to advance balloons and different microcatheters precisely in the middle LCX area in which the second “full moon”‐type plaque had previously been identified. Finally, we were able to place an atherectomy wire (Rotawire) in the LCX. After rotablation, intravascular ultrasound showed two severely calcified circular plaques, as previously detected by MSCT, with evidence of calcium fracture after atherectomy (Figure 4). The angiographic result was excellent (Figure 5).

## 4 DISCUSSION

Revascularization failure has often been linked to CTO calcifications, which either preclude the guide wire from crossing the lesion or facilitate balloon ruptures during inflation. This case shows how crucial is the role of MSCT in detecting complex lesions such as “full moon” plaques for CTO‐PCI, as demonstrated in literature.^1^, ^2^, ^3^

Over the years, despite development of new materials, new antegrade and retrograde approach techniques and increased expertise of the operators, the current success rate of CTO‐PCI is still not 100%. Calcification and particularly its disposition around the vessel is a key‐point in CTO‐PCI. A “full‐moon” plaque is an index of severe procedural complexity and a possible predictor of the need for atherectomy to achieve procedural success. Our case shows how this could help physicians plan the procedure properly and arrange for atherectomy; therefore, increasing the success rate of CTO‐PCI, especially in centers just starting a CTO program. Further larger studies are needed to confirm this hypothesis.

## AUTHOR CONTRIBUTIONS

**Giuseppe Panuccio:** Conceptualization; data curation; formal analysis; investigation; validation; visualization; writing – original draft; writing – review and editing. **Carsten Skurk:** Visualization. **Ulf Landmesser:** Conceptualization; methodology; supervision; visualization. **Youssef Abdelwahed:** Conceptualization; data curation; formal analysis; investigation; methodology; supervision; writing – original draft; writing – review and editing.

## FUNDING INFORMATION

This research received no specific grant from any funding agency in the public, commercial, or not‐for‐profit sectors.

## CONFLICT OF INTEREST STATEMENT

Nothing to declare.

## ETHICS STATEMENT

Not required.

## CONSENT STATEMENT

Written informed consent was obtained from the patient to publish this report in accordance with the journal's patient consent policy.

## Data Availability

The data that support the findings of this study are available from the corresponding author upon reasonable request.
